# New magnetic anomaly map for the Red Sea reveals transtensional structures associated with rotational rifting

**DOI:** 10.1038/s41598-022-09770-0

**Published:** 2022-04-06

**Authors:** Ran Issachar, Jörg Ebbing, Yixiati Dilixiati

**Affiliations:** Institute for Geosciences, Kiel University, Kiel, Germany

**Keywords:** Geodynamics, Geomagnetism, Geophysics, Tectonics, Solid Earth sciences

## Abstract

The Red Sea is a modern analogue for studying continental break-up. Particularly, the Red Sea shows along-strike variability in the architecture, magmatism and associated style of rifting. In order to study these variabilities, continuous geophysical data that cover the entire length of the basin is desired. Our study aims to produce a continuous, reliable and robust magnetic anomaly map for the Red Sea. We present a new magnetic anomaly map for the Red Sea, derived from re-processing of shipborne data, merged and conformed to a recent satellite model, LCS-1. The new magnetic map reveals prominent patterns of magnetic anomalies in sub-perpendicular directions to the Red Sea, with a northward increase in obliquity. We provide further analysis for the magnetic data and associate sets of magnetic trends with transtensional basement structures. Directional analysis suggests a gradual increase in shear component along the Red Sea. The magnetic trends are coaxial with independent indicators of finite and instantaneous strains, and thus implies that these structures and their variability are related to the kinematic framework of the rift. We discuss the consequences of rifting close to the Euler pole, i.e. rotational rifting, and argue that both passive and active forces can explain an increased along-strike transtension, and accordingly the associated variability along the Red Sea.

## Introduction

**Figure 1:**
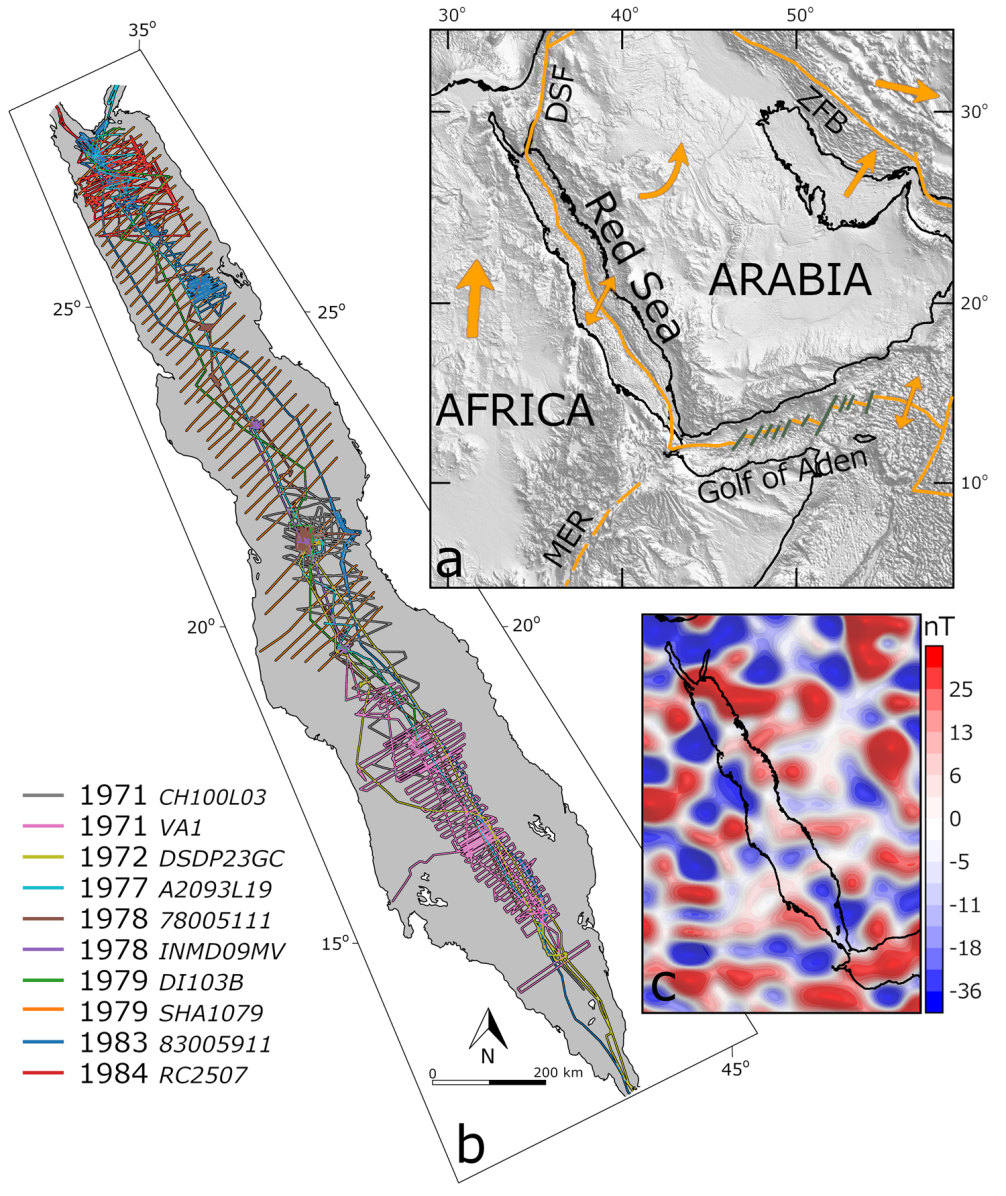
(**a**) Shaded relief map and regional tectonic settings (after Stern and Johnson, 2010)^72^ of the Red Sea area. (**b**) Track lines data used in this study. (**c**) Total magnetic field anomaly from satellite derived magnetic model, LCS-1.

The Red Sea is a young rift system, which encompass the separation of Arabia from Africa^1^ (Fig. 1a). It is considered as a modern analogue and a prime locality to study final stages of continental break-up and incipient seafloor spreading, as these are currently occurring in the Red Sea^2–6^. However, many aspects of its structure are still unknown or under debate^7^. In particular, its crustal structure and associated style of rifting appear to vary along its strike, where in the southern parts rifting is more developed and syn-rift volcanism is more abundant than in the northern parts^8^. Some authors attributed these differences to the proximity to a magmatic source within the Afar region^9,10^ or to different stages of rifting^11^. Yet, a major difficulty in the study of the Red Sea is the scarcity of robust geophysical data that cover the entire length of the basin^7^. Recent global compilations and satellite missions allow to study the Red Sea as one unit, and thus, to better compare its different structural regimes. For example, the availability of gravity models derived from satellite altimetry, global bathymetrical compilations, earthquake catalogs, digital elevation models etc. Magnetic anomalies can provide valuable knowledge of shallow crustal structures^12^, enabling geological interpretation in varied tectonic environments (e.g., East African Rift System^13,14^), complementary to the other geophysical data sets. However, a missing is a high-resolution and consistent magnetic anomaly map that covers the entire length of the Red Sea. Previously, high-resolution magnetic surveys were used to study the detailed crustal setting in specific regions within the Red Sea, e.g.^15^. Global magnetic compilations cover the Red Sea as well, e.g. EMAG2^16^ or WDMAM^17^, but these are less reliable for local regions of sparse and old data due to the automatic merging process. Even though, previous interpretations of global compilation derived magnetic and gravity anomalies in the Red Sea recognized sub-perpendicular lineaments and interpreted them as NE-SW and N-S trending faults^7,18^.

The primary goal of this study is to produce a consistent and reliable magnetic anomaly map for the entire length of the Red Sea by utilizing a compilation and analysis for shipborne and satellite magnetic data. By manually examining available track line measurements of past surveys, re-processing, leveling and conforming them to recent satellite magnetic model, we managed to construct a consistent and reliable magnetic anomaly map that covers the entire length of the basin. Grids with 5 km and 1 km cell size, as well as 10 km spaced cross-sections are available to download (see link at the Data and Methods section). Furthermore, we perform some simple field analysis and compare them with other geological and geophysical evidence. We discuss the implications of the results and draw attention to the role of the unique kinematic framework of the Red Sea, in-which rifting is occurring close to the Euler pole, i.e. ‘rotational rifting’. By this we contribute another layer of knowledge, strengthening previous insights regarding the tectonic evolution of the Red Sea.

## Geological setting

The Red Sea basin is a ~ 2000 km long rift, which cuts the Precambrian Arabo-Nubian shield, and accumulates ~ 200–300 km of extension. Together with the rifts in the Gulf of Aden and in the Afar triangle, it constitutes the ongoing separation of Arabia from Africa, a process which started around 30 Ma^1^ (Fig. 1a). Continental rifting began simultaneously along the entire Red Sea around ~ 23 Ma, preceded by intensive volcanism, mainly within the Arabian side^19,20^. Syn-rift magmatism is unevenly distributed along the Red Sea, where marginal magmatism is only recognized along its southern regions. Accordingly, the Red Sea was considered to incorporate both magma-rich margins in the south, and magma-poor margins in the north, however this subject is not yet clear^8^.

The Red Sea has a distinctive morphology, including elevated rift flanks, narrow marginal shelves and coastal plains, a wide main trough and a deep and narrow axial trough. Above the main trough and the coastal plains, a thick sedimentary cover (~ 6500 m) includes evaporitic sequences^1^ that masks the basement structures. In the south, the coastal plains are rather wide and the axial trough is deep and sharp, floored by common oceanic tholeiitic basalts^21–23^. Three magnetic stripes are recognized along the axis of the basin^18^, between latitudes 15.5° and 17.5°, indicating the development of organized seafloor spreading center at 4.6 Ma, and its gradual northward propagation. In the northern and central regions, the coastal plains are narrow and has a step wise architecture^24^, and a series of axial deeps are interrupted by giant submarine salt flows^25^. The deeps are floored by tholeiitic basalts and expose typical (ultra)slow-spreading mid-ocean ridge features, including volcanos and overlapping spreading centers^26^.

The development of seafloor spreading in the Red Sea that is associated with magnetic stripes, is considerably lagging behind that of the Gulf of Aden (which began at 16 Ma^27^), although rifting initiated almost concurrently along these segments^1^. Nevertheless, the nature of the crust in the Red Sea is a matter of long debate, where some authors favor vast dispersion of oceanic crust^10^ while others argue for a stretched continental crust^28^. However, due to the absence of available modern seismic data and deep boreholes that penetrate the thick sedimentary cover, it is not yet clear if most of the Red Sea has oceanic or continental crust. Moreover, dense seismic networks are only available along the Arabian margins and in the Afar triangle, and thus tomographic^29,30^ and receiver function^31,32^ models lack the resolution to infer the architecture of the lithosphere beneath the Red Sea.

## Results

### The new magnetic anomaly map

After careful evaluation, we found the magnetic data of 10 marine surveys, conducted between 1971 and 1984, reliable for further processing (Fig. 1b; see “Data and Methods” for more information). The distribution of the data sets allows a coast to coast coverage above latitude 20°, whereas below latitude 20° the coverage is mainly restricted to the middle section of the basin. The data sets were re-processed, levelled and conformed to satellite magnetic model (LCS-1; Fig. 1c), and the long wavelengths part between spherical harmonic degrees 15 to 110 (above ~ 360 km) was replaced (see “Data and methods” section for further details).

**Figure 2:**
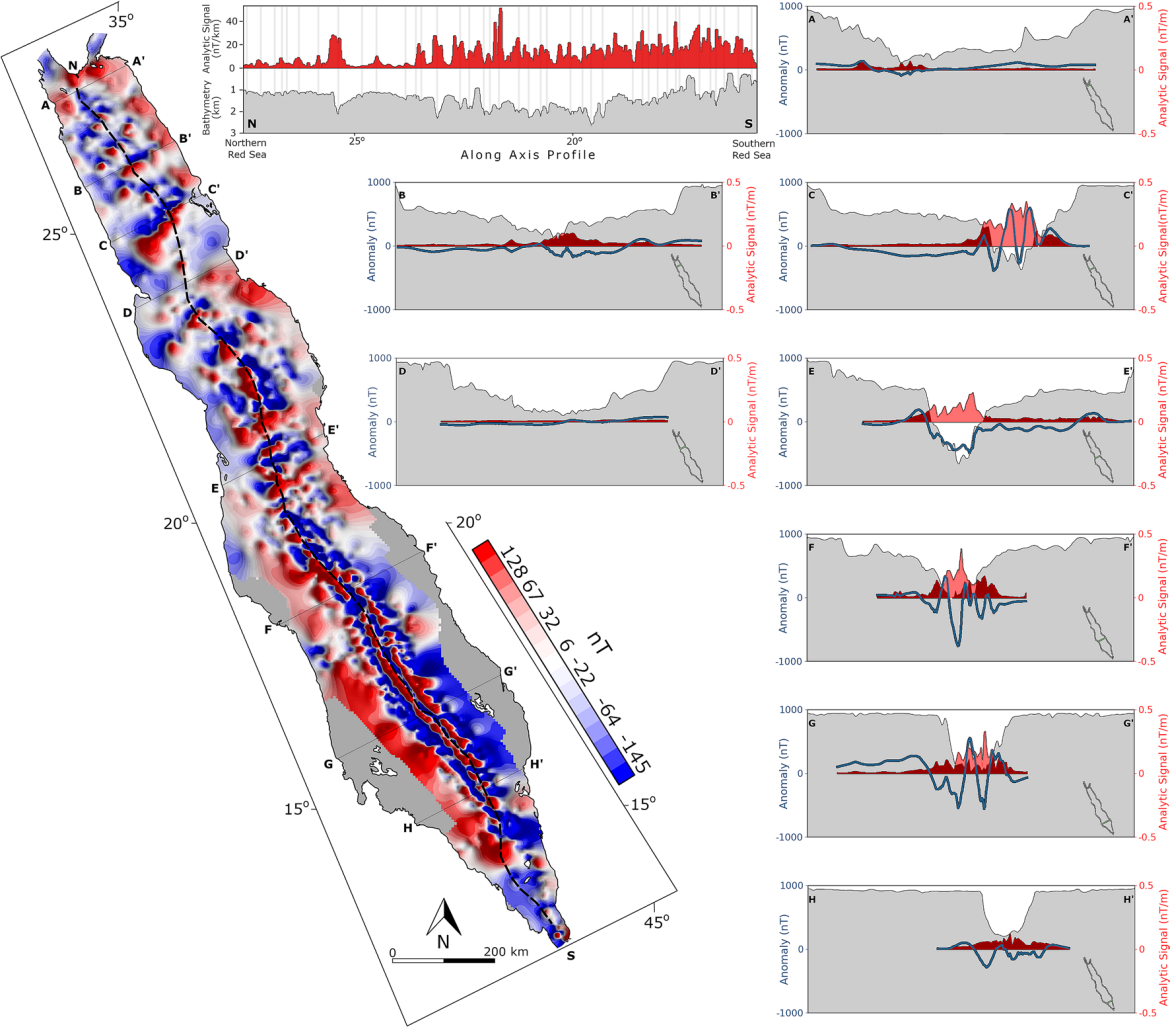
New magnetic anomaly map for the Red Sea and representing cross-sections. The map was produced from reprocessed track line data, conformed to satellite magnetic model (LCS-1). The magnetic data was gridded using minimum curvature algorithm with 5 km^2^ grid cell. Dashed black line marks the Red Sea axis. Cross-sections show: bathymetry (grey; 0 to 3 km), magnetic anomaly (blue), analytic signal (red).

The new magnetic anomaly map of the Red Sea shows a continuous and consistent magnetic anomaly field along the entire length of the basin, with significantly improved resolution in comparison with available global compilations (Fig. 2). Perpendicular cross-sections (Fig. 2A–H) indicate stronger magnetic signal above the axial trough, with higher values in the southern regions and above the bathymetric deeps. Along axis profile indicates a general correlation between bathymetry and magnetic strength, with analytic signal peaks above the bathymetric deeps (N-S section in Fig. 2). Magnetic stripes are well recognizable between latitudes 15° and 20° and are associated with young seafloor spreading^18^. In addition, basin parallel lineaments are observed along the edge of the coastal plains, associated with common rift normal fault^24^. Patterns of anomalies with sub-perpendicular orientations to the Red Sea are noticeable, mostly in the central and northern parts, with a generally northward increased obliquity (Fig. 2).

### Magnetic trends and directional analysis

**Figure 3:**
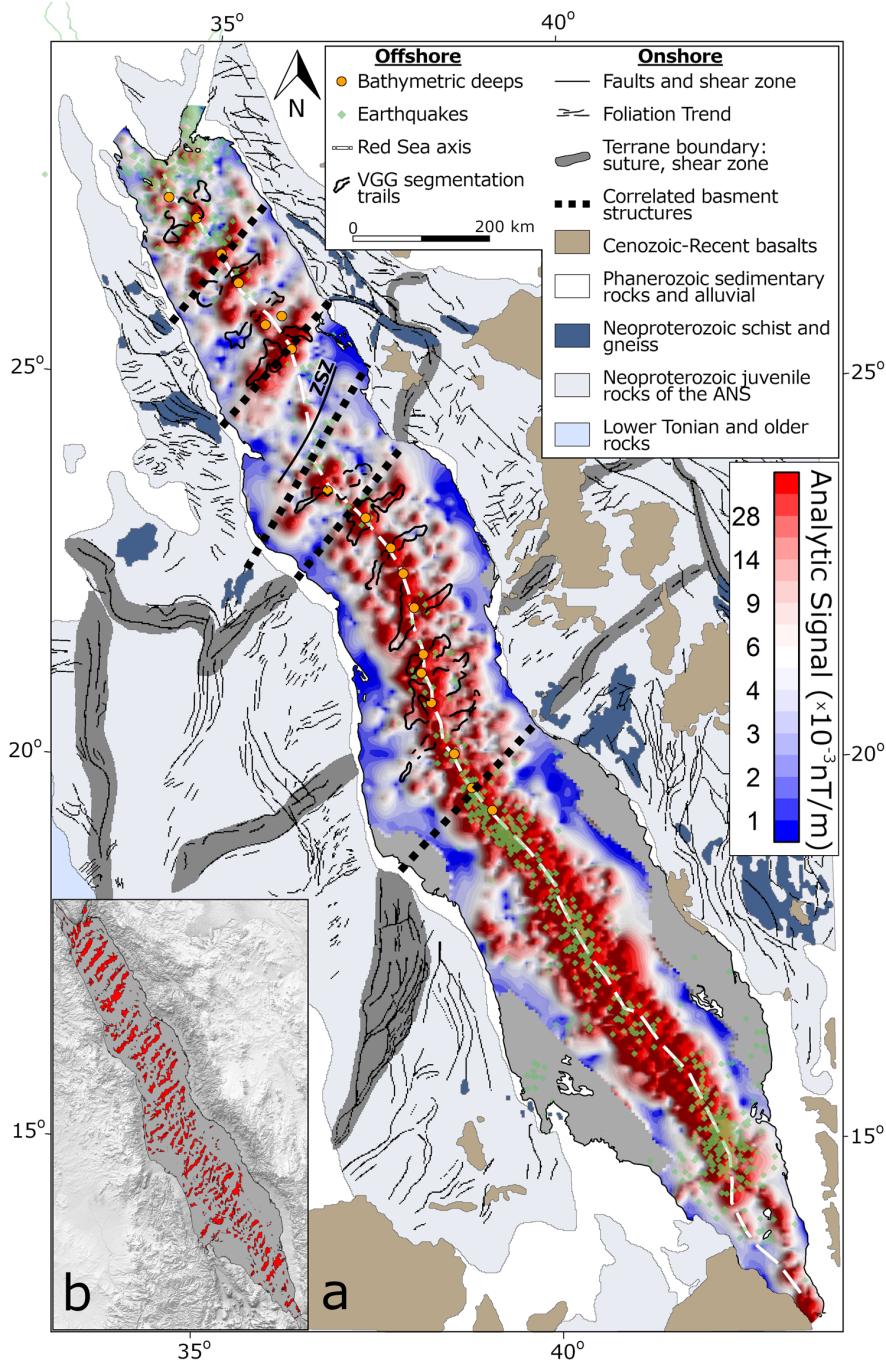
(**a**) Analytic signal of magnetic anomalies. The map shows bathymetry deeps (orange circles), earthquake locations (> 3.5 ML; green diamonds), mapped Vertical Gravity Gradient segments^10^ (black contours) and the axis of the Red Sea (dashed white line). Onshore geology and correlated basement structures (tick black dashed lines; after Stern and Johnson, 2018)^48^. ZSZ is the Zabargad Shear Zone. (**b**) Edge detection filtered analytic signal data using “neon” algorithm (Gimp.org).

**Figure 4:**
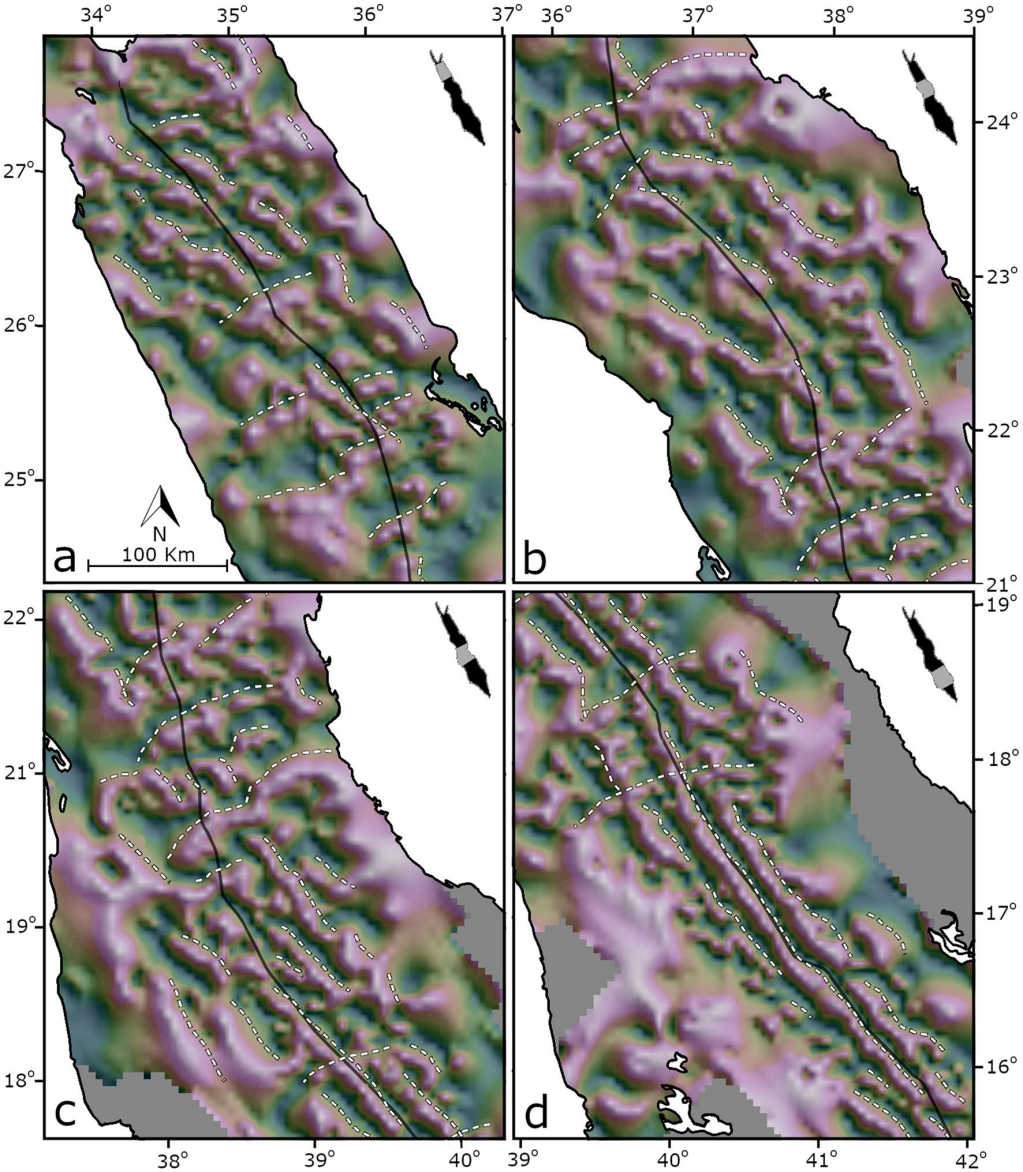
Tilt‐angle derivative map of magnetic anomalies, projected on a shaded relief. Purple colures represent positive angles and green colors represent negative angles. Black lines mark the Red Sea axis. White dashed lines highlight lineaments.

The analytic signal and the tilt‐angle derivative operators allow us to highlight magnetic trends, as they don’t have an inherent dipole nature, they eliminate low-latitude artifacts and present the anomalies directly above their sources (see “Data and methods” section). Magnetic trends with basin sub-perpendicular orientations are very prominent in the analytic signal map (Fig. 3). These show patterns of anomalies orientated sub-perpendicular to the Red Sea, extending over the main trough and coastal plains with lengths of tens to hundreds of kilometers, and even show a nearly coast to coast extent (Fig. 3). The edge detection filtering indicates that these trends are abundant throughout the entire length of the basin (Fig. 3b). Accordingly, we refer these trends as ‘regional’. The regional trends are also recognized in the in the tilt‐angle derivative map (Fig. 4).

**Figure 5:**
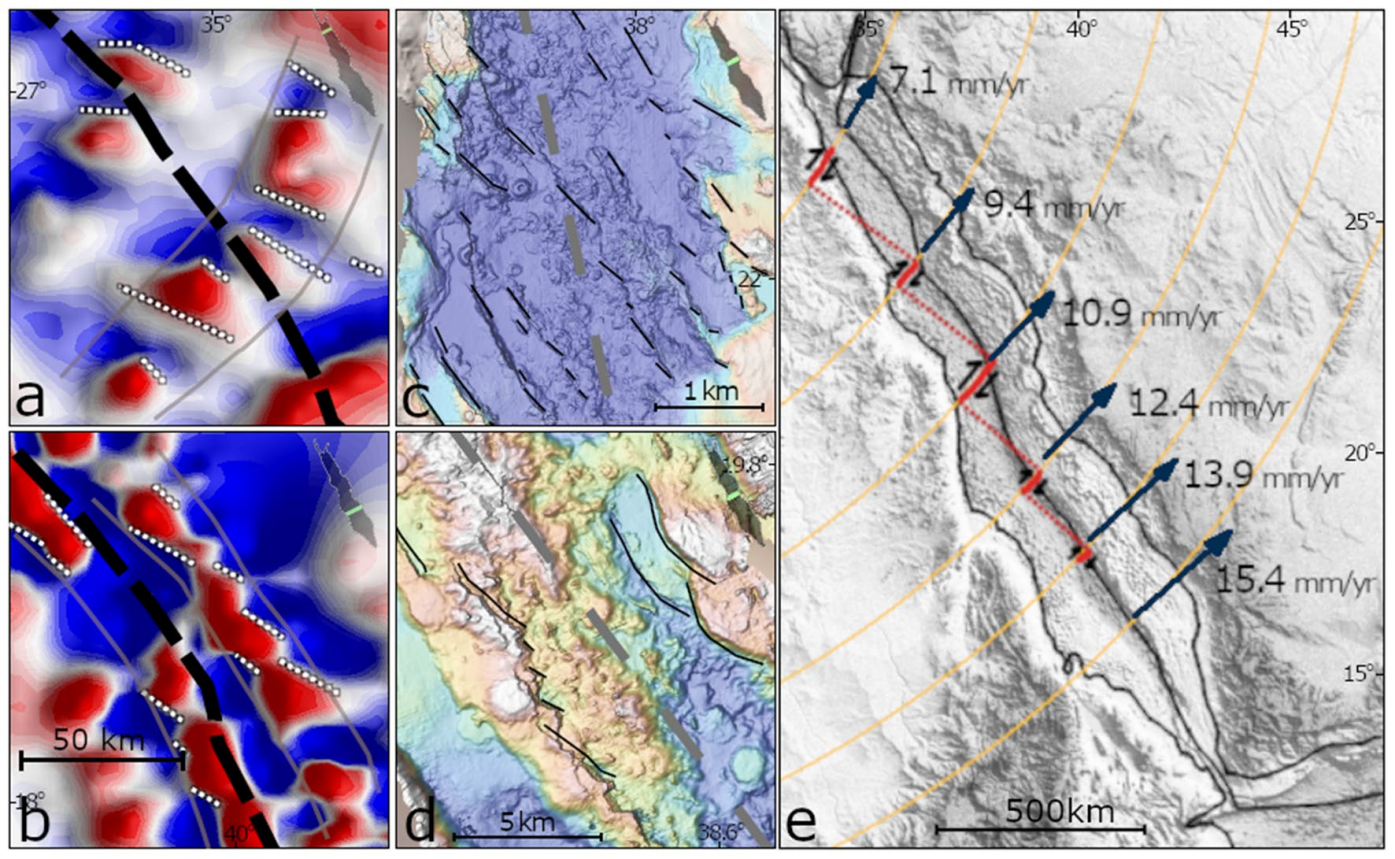
(**a**,**b**) Two examples of ‘local’ trends of magnetic anomalies from the northern (**a**) and southern (**b**) regions. Patterns of lineated anomalies forming en échelon structures. Black dashed lines mark the Red Sea axis. See Fig. 2 for color bar. (**c**,**d**) High resolution bathymetry, retrieved from Augustin et al. (2016)^73^ showing en échelon structures (black) oblique to the Red Sea axis (grey dashed lines). (**e**) Modeled velocities (blue arrows) along the axis of the Red Sea (after ArRajehi et al., 2010)^49^, and, small circles (yellow) around estimated Euler pole (after Schettino et al., 2016)^18^, suggesting dextral transcurrent flow sub-perpendicular to the rift axis, with a northward increasing shear and obliquity.

**Figure 6:**
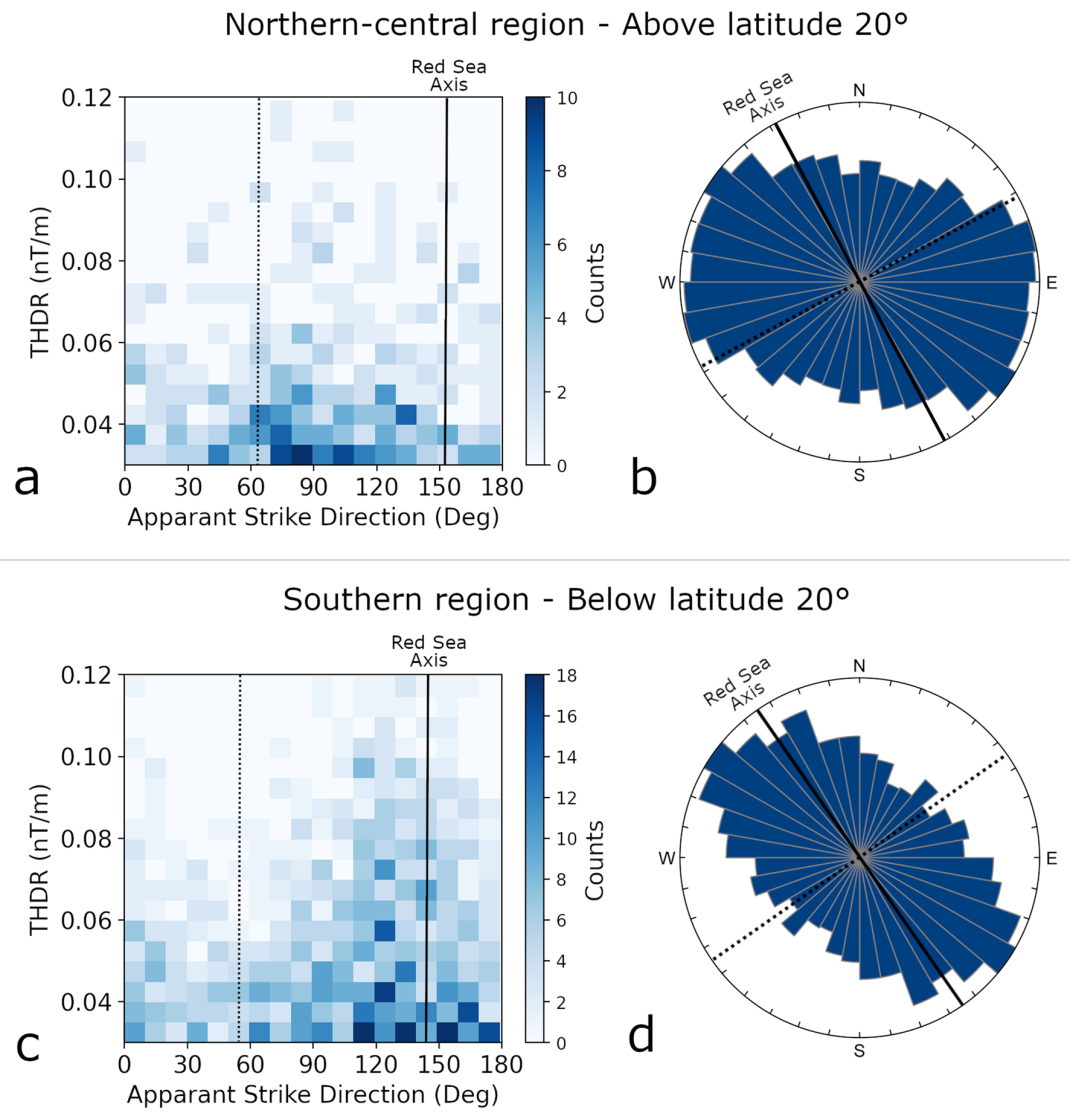
Apparent strike analysis of magnetic anomalies. 2-D histograms (**a**,**c**) shows the distribution of the total horizontal derivative (THDR) and azimuth of apparent strikes. The apparent strike is the direction perpendicular to the horizontal gradient. Ros diagrams (**b**,**d**) shows frequencies of apparent strike directions, produced with 10° bins and equal distance projection. Solid black line marks the average orientation of the Red Sea axis and its perpendicular direction (dashed black line).

In addition, we also recognize ‘local’ trends, of smaller scales, which include lineated anomalies in oblique directions to the axis of the Red Sea. Figure 5 shows two examples of the ‘local’ trends. A series of dipolar anomalies from the northern Red Sea, lineated in a NW–SE direction, oblique to the Red Sea axis, forming a circular en échelon structure (grey lines) (Fig. 5a). Another example from the southern Red Sea show oblique lineated anomalies that form en échelon structure along a pattern sub-parallel to the Red Sea (Fig. 5b). The apparent strike analysis indicates that the local trends are abundant along the entire length of the Red Sea (Fig. 6). In the northern-central regions, high THDR (Total Horizontal Derivative, see “Data and methods” section for an explanation) are mostly dispersed between azimuths of 060° and 140° (Fig. 6a), and peaks in the rose diagram are recognized in NW–SE and ENE–WSW directions (Fig. 6b). In the southern region, high THDR values are mostly dispersed between azimuths of 110° and 160° (Fig. 6c), and the rose-diagram shows a single peak in NW–SE direction (Fig. 6d).

## Discussion

### Origin of the magnetic trends

The new magnetic map reveals the abundance of regional and local magnetic trends sub-perpendicular to the Red Sea. The orientation of the regional and local magnetic trends correlates with dextral transtensive structures, where the regional trends denote their main orientations and the local trends represent internal secondary oblique structures. In these kind of settings oblique secondary structures and en échelon lineaments are abundant^33,34^. Several independent observations support the association of the magnetic trends with transtensional structures: (1) The signature of the Zabargad Shear Zone in the magnetic data with major magnetic highs along its edges (Fig. 3). The Zabargad Shear Zone is a NE-SW transverse structure, offsetting the Red Sea axis by ~ 100 km, was previously associated with transtensive tectonics and with the formation of the Mabahiss pull-apart^35,36^. (2) The magnetic trends have similar orientations to a transtensional structure in the Brothers Islands region (26.2°N, 34.5°E), observed in recent seismic data, including a ~ 20 km^2^ transtensive structures with ENE trending dextral main transcurrent faults and NW secondary normal faults cutting basement rocks^37^. We note, that the Brothers Islands are associated with magnetic peak in our magnetic map, and, that the regional and local magnetic trend are coaxial with the main and secondary mapped faults, respectively. (3) The magnetic trends have similar orientations to previously recognized transverse structures in the central regions of the Red Sea^38^. (4) The local magnetic trends have similar orientation to en échelon faults recognized in high-resolution bathymetry in the axial trough (Fig. 5c,d).

Sub-perpendicular lineaments were also observed in gravity data^39^, which for some extent correlate with the magnetic trends (Fig. 3). These were interpreted by some authors as being related to major, ridge-crossing fault zones^7,18^. However, lack of evidence for first-order (transform) ridge offsets in the bathymetry (except to the Zabargad Shear Zone) and comparison with other (ultra)slow-spreading mid-ocean ridges suggest that these lineaments are related to segmentation trails of second-order (non-transform) offsets^10^. Second-order offsets, typical to (ultra)slow-spreading mid-ocean ridges, were also observed along the ridge axis from high-resolution bathymetry^26^. These indicate right lateral sense of offsets, in accordance with our interpretation for the magnetic trends.

Nevertheless, the magnetic trends are not prominent in the bathymetry. The lack of bathymetric expression could be attributed to decoupling effect of weak salt layers in the sediment column. The magnetic signal is mainly derived from basement rocks and intrusion because of their large iron content, whereas sediments usually have low magnetic susceptibility and thus negligible contribution to the magnetic anomalies^40^. A compilation of outcrop and sub-surface data suggests ~ 6500 m of sedimentary cover in the coastal plains and main trough of the Red Sea^1^, which is rich in evaporates (1 to 3 km thick) in addition to carbonates, shales and sandstones. Although several wells penetrated metamorphic and basaltic rocks, the sedimentary section seems to be poor in volcanic intrusions^7,41^. Mechanical decoupling due to halite rich evaporitic sequences may prevent basement deformation to affect the rocks above^42–46^. In this case, the bathymetry is mostly affected by halokinetics^9,47^. Thus, the magnetic trends, probably reflect basement structures, which has no prominent bathymetric expression due to the decoupling effect of the sediments cover.

### The role of rotational rifting

The magnetic trends are remarkably coaxial with onshore kinematic indicators inferred from reconstruction of pre-rift rock exposures of the Arabo-Nubian shield along the rift flanks^48^ (dashed black lines in Fig. 3). Moreover, these are also parallel to present‐day GPS directions measured along the Arabian flanks^49,50^, and to current plate kinematic flow lines estimated from onshore kinematic indicators^18^ (Fig. 5e). Moreover, the magnetic trends and the onshore kinematic indicators show a similar increased obliquity, from south to north along the Red Sea, in similar to the regional magnetic trends. Accordingly, our directional analysis suggests an increased shear in the northern-central regions, as the apparent strikes show larger sets and higher obliquity compared to the southern regions (Fig. 6).

The parallelism of the magnetic trends to finite and instantaneous strain indicators within the adjacent plates, implies that internal rift structures are linked to the kinematics of the associated plates. In this manner, the unique kinematic framework of the Red Sea, in-which rifting is occurring close to the Euler pole, i.e. ‘rotational rifting’, can explain the development of intra-basin dextral transtensive structures. Field observations^51–53^ and recent analogue models^54,55^ show that passive forces within rotational rifts can have a significant role in their development including oblique extensional faults and en échelon structures, second-order discontinuities, hampered and even halted rift propagation. Accordingly, by considering the estimated velocities and Euler pole location for the Red Sea, a dextral transcurrent flow is expected along the Red Sea, as rotation rates increase with the proximity to pole, i.e. the angular velocity is higher from south to north (Fig. 5e). As a result, internal rift transtensive structures would develop with increased obliquity and increased shear along the rift. In addition to passive forces, active forces are also suggested to contribute to the development of shear regimes along rotational rifts^56^. Numerical experiments showed that induced gravitational forces of asthenosphere doming lead to large regions of transcurrent stress regime after an initial phase of orthogonal extension^57^. These active transtensive forces are also expected to grow with the proximity to the Euler pole. In conclusion, during rotational rifting both passive and active forces contribute the development of intra-rift transtensive structures with increased shear along the rift. This in turn, can explain the variability in the style of rifting along the Red Sea and its uniqueness in comparison with non-rotational rifts.

### The nature of the crust

Another insight is the strong correlation between the amplitudes of the magnetic anomalies and bathymetry. Along the entire basin, the deep regions are characterized by high amplitudes of the analytic signal, whereas the shallow regions are characterized by low amplitudes (see the cross-sections in Fig. 2; more cross-sections are available in the online repository). This correlation could be explained by differences in the nature of the crust, and/or, by the presence of thick sedimentary cover in the main trough and coastal plains. The province of the active spreading center in the southern Red Sea, which is characterized by deep bathymetry and intensive seismic activity of neovolcanic origin^58^, is also characterized by high magnetic amplitudes (Fig. 3). This provides a clear indication that seafloor spreading along the axial trough is associated with high amplitude magnetic anomalies. Moreover, in the northern-central parts of the Red Sea, we recognize peaks in the analytic signal above each of the bathymetry deeps (Fig. 2), which were shown as regions of active volcanism^25,36,59^. Dredged samples from the Red Sea deeps indicate basaltic seafloor of tholeiitic composition, typical to slow spreading mid-ocean ridges^21–23,59^. These basalts are associated with high magnetization values due to large content of iron bearing minerals and fast cooling rates, and thus, are a source for high amplitude magnetic anomalies^40^. Nevertheless, the nature of the basement rocks beneath the sedimentary cover in the main trough and coastal plains is debatable. As many authors speculate hyperextended continental crust (melt-intruded extended continental crust)^4,28^, some authors argue that oceanic crust must be present within larger portions of the Red Sea^10,48^. Seemingly, the low amplitude of the magnetic signal above the main trough and coastal plains supports the hyperextended continental crust option, however previously suggested explanations could explain low amplitude anomalies of oceanic crust below thick column of sediments. These propose that if seafloor spreading is occurring under thick sedimentary cover, then high temperatures and enhanced hydrothermal alteration at the base of the sedimentary blanket would prevent the acquisition of strong magnetization of the oceanic crust^10,25,60,61^. These processes were shown to explain magnetic lows at specific locations within the present-day axial trough were massive slumps of sediments and evaporites are present^25^, however, it is not clear if this situation is realistic in earlier stages of the rift^28^. As the thermo-chemical conditions required to produce stable single-domain magnetite that would hold natural remanent magnetization are very fragile, it is difficult to speculate from the magnetic data the nature of the crust in the main trough and coastal plains. Nevertheless, we don’t recognize any evidence for oceanic crust under the sedimentary cover in the magnetic data.

## Conclusions

The new magnetic anomaly map of the Red Sea shows a continuous and consistent magnetic anomaly field along the entire length of the basin, with significantly improved resolution. The new magnetic anomaly map provides insights into the basement structure of the basin. ‘Regional’ and ‘local’ magnetic trends with sub-perpendicular orientations to the Red Sea are prominent and are associated with basement structures, developed during dextral transtensive stress field. The magnetic trends are coaxial with finite and instantaneous strain indicators and show increased obliquity along the Red Sea, from south to north, indicating increased shear accordingly. Passive and active forces, that are associated with rotational rifting explain the increased shear and the variability in the style of rifting along the Red Sea. These insights demonstrate that along-strike variability is inherent and significant in rotational rifts.

## Data and methods

### Conforming track line and satellite magnetic data

The Red Sea was intensively surveyed from the early 60’s up to mid-80’s. Large amounts of geophysical data was collected in specific regions within the Red Sea. We carefully examined different data sets (retrieved from the NGDC database) in order to identify reliable magnetic track line measurements. To evaluate the quality and reliability of the data we monitored the noise level by examining the ‘fourth difference’^62^ and rejected data with noise level exceeding 100 nT. We found that all surveys prior to 1971 are unreliable, and considered 10 surveys as reliable (Fig. 1b). Two surveys, SHA1079 and VA1, cover extended areas and are composed of parallel lines in perpendicular direction to the Red Sea axis. The SHA1079 dataset, collected by the British RRS Shackleton cruise in the winter of 1979, consist of 44 lines from the northern Red Sea down to latitude 20° with average line separation of 22 km. Total field measurements are not reported and the data has no tie-lines (lines of measurements that cross the survey lines), and thus, further processing was infeasible. Nevertheless, the reported data include diurnal correction from onshore base station and the overall quality of the data seemed very good. The VA1 dataset, collected by the German Valdivia cruise in 1971, includes more than 60 dense survey lines from latitudes 14.5° to 19.5°, mainly within the middle section of the basin (Fig. 1b). Line separation varies from 8 to 3 km with few tie-lines perpendicular to the survey lines. The other surveys are mostly focused at specific areas, however, include some long lines which helped in the leveling process of the surveys.

To merge the datasets, we preformed initial leveling using a statistical levelling approach^63^. In the first step, we calculated the magnetic residuals by subtracting the definitive magnetic reference field model (DGRF) from the total field measurements. For the SHA1079 dataset, the original measured values are not documented, as well as the processing that was performed. For that, we relied on the original corrections and minimized the disparities in the following steps. In the second step, we calculated a least-squares surface trend for the intersection errors, i.e. the difference between values at intersection points, and then removed that trend from the entire datasets. We regarded the SHA1079 dataset as a base level and leveled all the other datasets relative to it. After the initial levelling we gridded the datasets together with: (1) 5 km spacing, and, (2) 1 km spacing. Processing of magnetic data was preformed using Oasis montaj software package (Geosoft Inc.).

Further, to correct the long wavelength parts and to perform fine leveling of the datasets we conformed the gridded data to a satellite derived magnetic model (LCS-1). The LCS-1 model includes observations from CHAMP and Swarm missions, providing reliable wavelengths at mid-latitudes from wavelengths of 250 km^64^. As the maximum wavelength in each of the datasets is limited by the extent of the survey, the long wavelengths in the merged grid are expected to include errors. To overcome this difficulty, we adopted an equivalent layer approach to conform the datasets to a recent LCS-1 satellite magnetic model, calculated at ellipsoid height (Fig. 1c)^65,66^. The analysis indicated that wavelengths above ~ 350 km (spherical harmonic degrees 15 to 110) are not consistent and thus were replaced.

**Figure 7:**
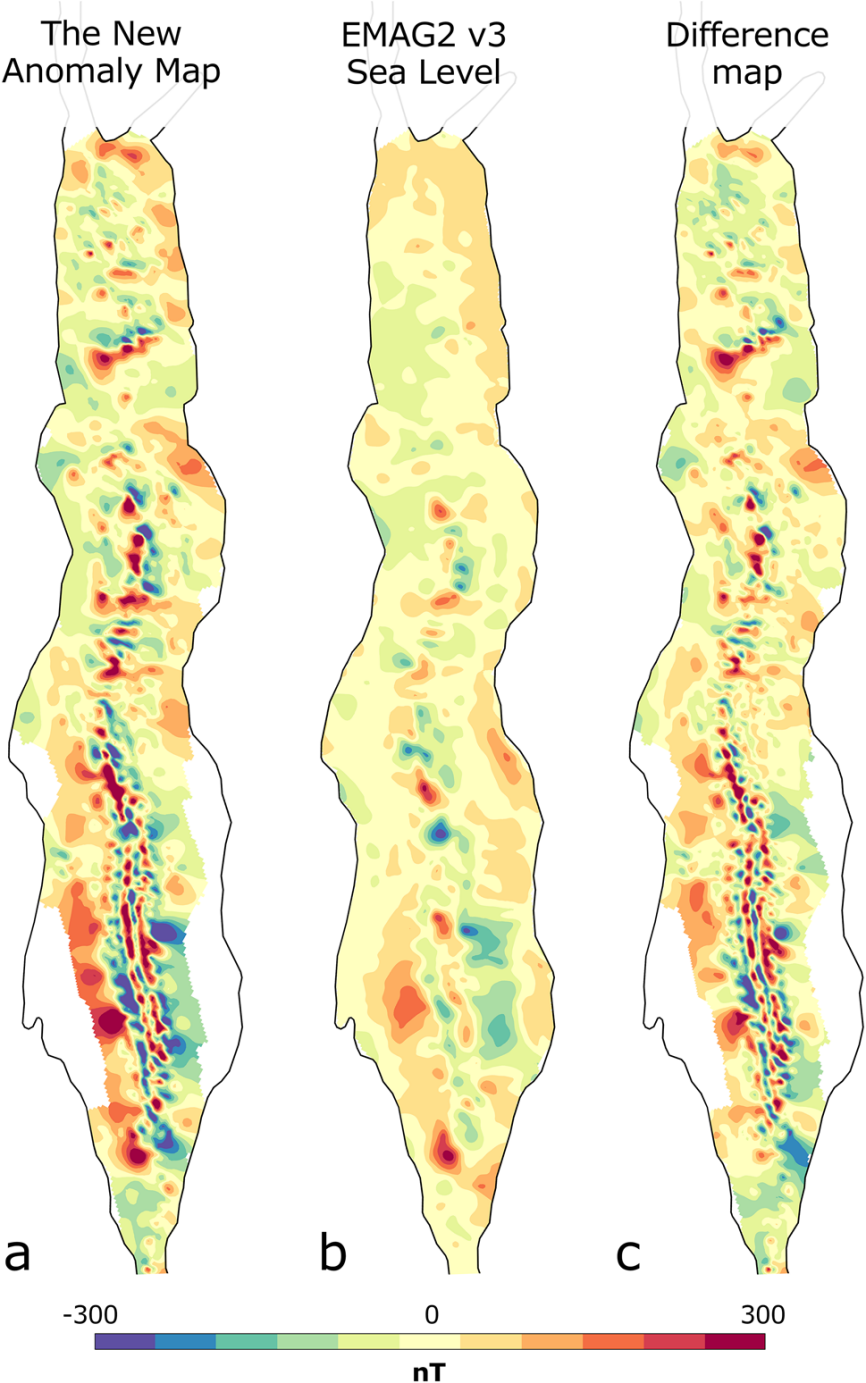
Comparison between (**a**) the new magnetic anomaly map produced in this study and (**b**) the EMAG2 v3 global compilation, and, the differences between them (**c**). The maps are presented with linear colormaps.

Figure 7 shows a comparison between the new magnetic anomaly map produced in this study and a global compilation derived map (EMAG2 version 3^67^). The new map shows significantly improved resolution and includes more details in short wavelengths, yet, the general structures agree well with the EMAG2 global compilation. The EMAG2 compilation has coverage near the coastal plains in the southern parts as it includes local and regional aeromagnetic surveys and compilations and satellite data. We limit our compilation to the area, where reliable track line data are available to us.

### Analysis of magnetic anomalies

Because the Red Sea is located at low magnetic latitudes (the geomagnetic inclination is 20° in the south and 40° in the north), direct interpretation of the anomalies is deceptive and may lead to false conclusions. The vector nature of the magnetic fields increases the complexity of anomalies from magnetic rocks, and these are usually seen as asymmetrical dipolar anomalies not necessarily centered above the source. For this, the anomaly field is often reduced to the magnetic pole, however, this process is less applicable in the current case because: (1) significant contribution from remanent magnetization is expected, (2) the different data sets acquired during a large time span, and, (3) the increased noise problem arises from north–south features would add uncertainties^68^. To overcome these difficulties, we adopted the ‘analytic signal’ approach, which ensures maxima above magnetic sources and produce anomaly shapes that are not affected by the direction of magnetization^69^. The analytic signal is calculated at each grid location from the three spatial derivatives of the anomaly field.

In order to enhance the edges associated with magnetic trends we applied a tilt‐angle derivative operator, which enhances shallow subsurface anomalies and short wavelength lineaments due to the normalizing effect of the tilt‐angle derivative operator^70^. The tilt‐angle derivative has the characteristic of being positive over a source and negative elsewhere and is calculated for each grid location from the three spatial derivatives of the anomaly field. Further, we applied transparency to the tilt‐angle derivative map and projected it on a shaded relief.

To further quantify the directional trends of the magnetic data, we analyzed the apparent strike of the magnetic anomalies^71^. The apparent strike is the strike of the horizontal gradient, i.e. the azimuth of the horizontal gradient plus 90°, and is utilized to infer directional insights from potential field data. At each grid point we calculated the horizontal derivatives of the anomaly field, and from these, the azimuth of the apparent strike and the magnitude of the horizontal gradient (THDR). We then plotted a two-dimensional histogram of THDR and apparent strike directions (Fig. 6a,c) and a rose-diagram for the apparent strikes with 10° bin width (Fig. 6b,d).

## Data Availability

Our new compilation grids and cross-sections can be accessed at: https://figshare.com/articles/dataset/Transcurrent_Regimes_During_Rotational_Rifting_New_Insights_from_Magnetic_Anomalies_in_the_Red_Sea/14743272. Magnetic track line data was retrieved from the National Centers for Environmental Information (NCEI) and is available at https://www.ngdc.noaa.gov/mgg/geodas/trackline.html. The satellite model LCS-1 can be accessed at https://www.space.dtu.dk/english/research/scientific_data_and_models/magnetic_field_models. Bathymetric data was retrieved from GEBCO Compilation Group (2020) GEBCO 2020 Grid (10.5285/a29c5465-b138-234d-e053-6c86abc040b9), available at https://www.gebco.net. High resolution bathymetry used in Fig. 5 is available at https://doi.pangaea.de/10.1594/PANGAEA.860374. Earthquake data includes events with magnitudes > 3.5 ML between 1980–2020, retrieved from the International Seismological Centre (2020), On-line Bulletin, 10.31905/D808B830. All the maps were produced using Oasis montaj software package (Geosoft Inc.).
